# Salicylic Acid and Water Stress: Effects on Morphophysiology and Essential Oil Profile of *Eryngium foetidum*

**DOI:** 10.3390/metabo14040241

**Published:** 2024-04-21

**Authors:** Sabrina Kelly dos Santos, Daniel da Silva Gomes, Vanessa de Azevedo Soares, Estephanni Fernanda Oliveira Dantas, Ana Flávia Pellegrini de Oliveira, Moises Henrique Almeida Gusmão, Elyabe Monteiro de Matos, Tancredo Souza, Lyderson Facio Viccini, Richard Michael Grazul, Juliane Maciel Henschel, Diego Silva Batista

**Affiliations:** 1 Postgraduate Program in Agronomy, Federal University of Paraiba, Areia 58397-000, Paraíba, Brazil; sabrinasks11@gmail.com (S.K.d.S.); danielsgea@gmail.com (D.d.S.G.); nessieazevedo@gmail.com (V.d.A.S.); julianemhenschel@gmail.com (J.M.H.); 2 Department of Agriculture, Federal University of Paraiba, Bananeiras 58220-000, Paraíba, Brazil; estephanni.dantas@academico.ufpb.br; 3 Department of Chemistry, Federal University of Juiz de Fora, Juiz de Fora 36036-900, Minas Gerais, Brazil; ana.flavia.p.o@hotmail.com (A.F.P.d.O.); richard.grazul@ufjf.edu.br (R.M.G.); 4 Department of Biology, Federal University of Juiz de Fora, Juiz de Fora 36036-900, Minas Gerais, Brazil; gusmaomoises@hotmail.com (M.H.A.G.); elyagro@gmail.com (E.M.d.M.); lyderson.viccini@ufjf.br (L.F.V.); 5 Postgraduate Program in Agroecology, Federal University of Paraiba, Bananeiras 58220-000, Paraíba, Brazil; tancredo.souza@ufsc.br

**Keywords:** abiotic stress, bioregulator, *Eryngium foetidum* L., secondary metabolites, wild coriander

## Abstract

The exogenous application of bioregulators, such as salicylic acid (SA), has exhibited promising outcomes in alleviating drought stress. Nevertheless, its impact on culantro (*Eryngium foetidum* L.) remains unexplored. Thus, the aim of this study was to assess how SA impacts the growth, morphophysiology, and essential oil composition of culantro when subjected to drought. To achieve this, culantro plants were grown under three different watering regimes: well-watered, drought-stressed, and re-watered. Additionally, they were either treated with SA (100 µM) or left untreated, with water serving as the control. SA application did not mitigate the effects of drought in biomass production but increased biomass, leaf number, leaf area, and photosynthetic pigments under well-irrigated and re-watered conditions. After a drought period followed by re-watering, plants recovered membrane integrity independently of SA application. Water stress and the exogenous application of SA also modulated the profile of essential oils. This is the first report about SA and drought affecting growth and essential oil composition in culantro.

## 1. Introduction

The exponential growth of the world population requires an increase in agricultural production to meet food needs; however, one of the great challenges faced by agriculture is the production losses caused by abiotic stresses, such as drought, temperature, salinity, and heavy metals [1]. Moreover, considering climate change predictions, the impact of these abiotic stresses on agriculture is expected to be greatly worsened in the future [1,2]. Drought stress, for example, negatively affects plant growth and productivity, also inducing metabolic, physiological, and biochemical changes [2,3]. For instance, drought decreases cell elongation and division; affects photoassimilate production and partitioning; and induces oxidative damage due to the overproduction of reactive oxygen species (ROS) [4,5]. Plants can induce defense mechanisms, such as stomatal closure and osmotic adjustment, to prevent the harmful effects of water stress [6,7]. Under drought stress, plants close their stomata to prevent water loss, which consequently reduces CO_2_ uptake, directly affecting the photosynthetic rate [8]. This impairment in gas exchanges induces changes in leaf metabolism, inhibiting photosynthesis [4,7,9]. Moreover, under drought stress, plants can induce the production and accumulation of osmoprotective molecules, such as amino acids, carbohydrates, proteins, and inorganic ions, to maintain cell turgor [10].

Considering the strong impacts of drought on crop productivity, it is imperative to develop strategies to overcome these negative effects. In this context, plant breeding, optimal crop management, and plant bioregulators have been extensively used [2]. Bioregulators are natural or synthetic plant growth regulators, essential for plant development and metabolism under normal and stressful conditions [11,12]. Plant bioregulators include auxin, gibberellin, abscisic acid, cytokinin, salicylic acid (SA), nitric oxide, methyl jasmonate, paclobutrazol, and ethephon, among others [2,13,14].

SA is a phenolic compound naturally synthesized by plants and can also be applied exogenously via foliar or seed pretreatment [15,16,17]. SA regulates plant growth and development and plays an important role in defense against abiotic stresses, inducing local and systemic acquired resistance [18,19]. The application of SA regulates endogenous hormone levels [20] and modulates growth, the activity of antioxidant enzymes such as peroxidase, catalase, ascorbate peroxidase, and the production of secondary metabolites such as phenols and flavonoids [21,22]. In addition, SA increases carbon dioxide assimilation and chlorophyll content [23].

*Eryngium foetidum* L., popularly known as culantro and wild coriander, is a perennial herbaceous species native to Central America that belongs to the Apiaceae family [24]. Culantro resembles coriander (*Coriandrum sativum*) due to its strong aroma; however, it has a more resistant nature and a longer shelf life [25]. It is an aromatic plant, being used as medicine in China, India, Vietnam, Mexico, and the Amazon region [26]. In addition, secondary metabolites produced by culantro can be used against *Leishmania tarentolae*, *Leishmania donovani*, fungi, and bacteria such as *Bacillus cereus* and *Staphylococcus aureus* [27,28,29]. The essential oil of culantro is rich in aromatic and aliphatic aldehydes, with (2E)-2-dodecenal, also known as eryngial, being the predominant constituent, which is responsible for the aroma and flavor of this species [26,30]. To test the hypotheses that SA can modulate the profile of essential oils in *E. foetidum*, and that its exogenous application can mitigate water stress in this species, the aim of this study was to assess how SA influences the growth, morphophysiology, and essential oil composition of culantro plants when subjected to water stress.

## 2. Materials and Methods

**Experimental location.** The experiment was conducted from January to May 2022 in a greenhouse covered with transparent film, situated in the experimental area of the Seedling Production Laboratory at the Center for Human, Social, and Agrarian Sciences of the Federal University of Paraiba (CCHSA/UFPB) in Bananeiras, PB, Brazil. The coordinates of the location are 6°45′ S, 35°38′ W, with an elevation of 526 m. The methodology employed in this study is derived from the previous work of our research group [31].

**Irrigation and salicylic acid treatments.** Culantro (*E. foetidum* L.) plants were grown and, 50 days after sowing (DAS), subjected to these irrigation levels: 80% bag capacity (BC)—well-watered, and 40% BC—drought. Then, they were re-watered after twelve days of drought, according to Santos et al. [31]. Moreover, plants were leaf-sprayed with water (control) or 100 µM SA using hand sprayers (Bestfer, Duque de Caxias, Brazil) every six days. Leaves were sprayed until completely wet (12 mL per plant).

**Morphophysiological analysis.** At 102 DAS, growth, photosynthetic pigments, gas exchanges, and chlorophyll *a* fluorescence were measured. Leaf area, number of leaves, specific leaf area, and root length were quantified by image analysis utilizing the software ImageJ version 1.53k [24,31]. Shoots and roots were oven-dried at 65 °C until they reached a constant weight to determine their dry weight, shoot/root ratio, and total biomass. Gas-exchange and light-response curves of photosynthesis were measured with an open-flow gas-exchange infrared gas analyzer (IRGA, LCpro-SD Portable Photosynthesis System, ADC BioScientific, Hoddesdon, UK) on fully expanded leaves of five plants per treatment between 8:00 a.m. and 10:00 a.m., with the conditions previously defined by Santos et al. [31]. The net photosynthetic rate (*A*, µmol CO_2_ m^−2^ s^−1^), stomatal conductance (*g_S_*, mol H_2_O m^−2^ s^−1^), internal CO_2_ concentration (*Ci*, mmol CO_2_ mol^−1^ air), transpiration rate (*E*, mmol H_2_O m^−2^ s^−1^), water use efficiency (*A*/*E*), carboxylation efficiency (*A*/*Ci*), dark respiration (Rdark, µmol m^−2^ s^−1^), apparent quantum yield (mol mol^−1^), light compensation point (LCP, µmol m^−2^ s^−1^), maximum gross assimilation rate (Amax, µmol m^−2^ s^−1^), and light saturation point (LSP, µmol m^−2^ s^−1^) were determined. Photosynthetic pigment concentration (chlorophyll *a*, chlorophyll *b*, and total carotenoids) was determined according to Wellburn [32], with modifications proposed by Santos et al. [33]. Electrolyte leakage was quantified according to Bajji et al. [34] and Santos et al. [31], to assess tissue death in response to drought stress.

**Microextraction and qualitative analysis of essential oils.** Approximately 500 mg of leaves were collected and stored at −18 °C in test tubes with a screw cap, following Castro et al. [35], and the microextraction was performed following Santos et al. [31]. The qualitative analysis of essential oils was carried out on a gas chromatographer coupled to a mass spectrometer (GCMS-QP2010 Plus; Shimadzu, Kyoto, Japan) and an Rtx-5MS^®^ column (Restek, Bellefonte, PA, USA) of 30 m × 0.25 mm, with three technical replicates and running conditions according to Santos et al. [31]. The compounds were identified by comparing mass spectra to the NIST 9.0 database (correlation ≥ 95%) and confirmed with the Kováts retention index.

**Experimental design and statistical analysis.** The experimental design was completely randomized, using a 2 × 3 factorial scheme (SA application × water condition) with ten replicates, each consisting of one bag containing one plant. Data underwent normality and homogeneity tests (Shapiro–Wilk and Bartlett, respectively), followed by an analysis of variance and Tukey’s test (*p* ≤ 0.05), using Genes software version 2015.5.0 [36]. Growth parameters and essential oil profiles underwent multivariate analyses. Treatment distances were assessed through canonical discriminant analysis in a three-dimensional scatter plot. Treatments were grouped using the Tocher optimization method and Mahalanobis’ generalized squared interpoint distance (D^2^). Variable contributions to treatment discrimination were quantified using the Singh [37] criterion.

## 3. Results

**Figure 1:**
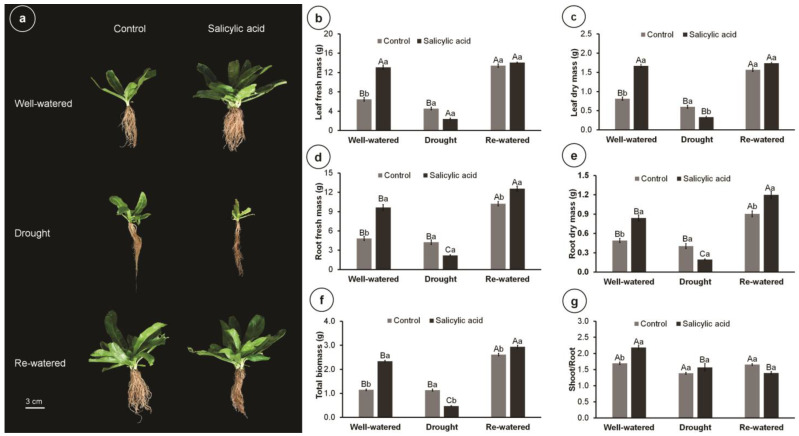
Biomass production and partitioning of 102-day-old culantro plants treated with water (control) or salicylic acid and grown under different water levels (80% BC—well-watered; 40% BC—drought; and re-watered after 12 days of water restriction). Values represent means ± standard error (*n* = 6). Capital letters compare water levels within each salicylic acid treatment, and lowercase letters compare control and salicylic acid treatment within each water level (Tukey’s test; *p* ≤ 0.05). (**a**) plant phenotype; (**b**) leaf fresh mass; (**c**) leaf dry mass; (**d**) root fresh mass; (**e**) root dry mass; (**f**) total biomass; (**g**) shoot/root ratio.

The highest plant growth was obtained under re-watered and well-watered conditions, while drought limited culantro development (Figure 1a). Without SA application, re-watering doubled the leaf fresh mass, root fresh mass, and total biomass, with increments of 108.61%, 111.38%, and 127.89%, respectively, compared to well-watered plants (Figure 1b,d,f). Re-watering also increased the leaf dry mass and root dry mass of control plants compared to the well-watered condition (Figure 1c,e). In well-watered plants, SA application increased leaf fresh mass, root fresh mass, leaf dry mass, root dry mass, and total biomass by 102.98%, 99.37%, 105.51%, 72.04%, and 103.79%, respectively. Similarly, SA treatment increased the root fresh and dry mass and total biomass compared to the control in re-watered plants. By contrast, SA decreased the leaf dry mass and total biomass of culantro compared to control plants. Water conditions did not affect the biomass allocation of control plants; however, compared to the control, SA increased the shoot/root ratio of well-watered plants (Figure 1g).

**Figure 2:**
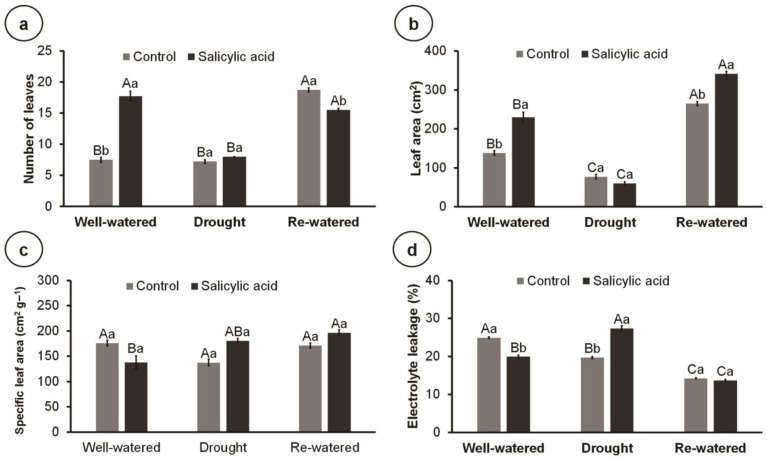
Number of leaves, leaf area, specific leaf area, and electrolyte leakage of 102-day-old culantro plants treated with water (control) or salicylic acid and grown under different water levels (80% BC—well-watered; 40% BC—drought; and re-watered after 12 days of water restriction). Values represent means ± standard error (*n* = 6). Capital letters compare water levels within each salicylic acid treatment, and lowercase letters compare control and salicylic acid treatment within each water level (Tukey’s test; *p* ≤ 0.05). (**a**) number of leaves; (**b**) leaf area; (**c**) specific leaf area; (**d**) electrolyte leakage.

Drought decreased the leaf area of control plants compared to well-watered and re-watered conditions (Figure 2a,b). On the other hand, SA increased the number of leaves by 136.67% and the leaf area by 66.08% compared to control plants under well-watered conditions (Figure 2a,b). Similarly, SA increased the leaf area of re-watered plants by 28.75% compared to the control. Water conditions did not affect the specific leaf area of control plants; however, in SA-treated plants, re-watering increased the specific leaf area compared to well-watered conditions (Figure 2c). Intriguingly, drought reduced the electrolyte leakage of control plants compared to well-watered conditions (Figure 2d). On the other hand, SA reduced electrolyte leakage in well-watered plants but increased it in drought-stressed plants. In turn, re-watering reduced electrolyte leakage independently of SA application (Figure 2d).

**Figure 3:**
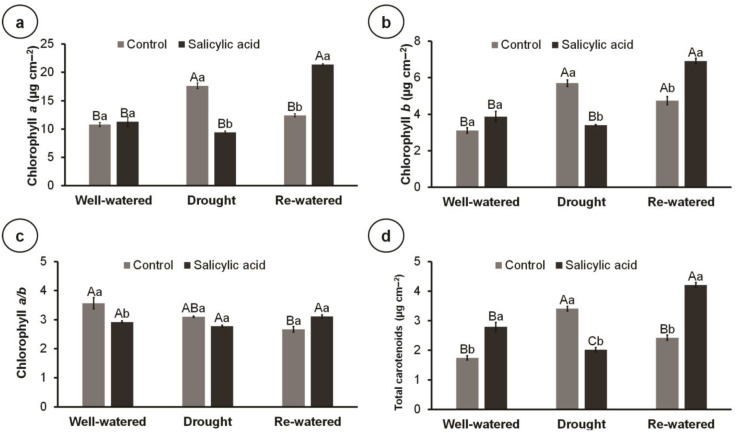
Photosynthetic pigments of 100-day-old culantro plants treated with water (control) or salicylic acid, and grown under different water levels (80% BC—well-watered; 40% BC—drought; and re-watered after 12 days of water restriction). Values represent means ± standard error (*n* = 6). Capital letters compare water levels within each salicylic acid treatment, and lowercase letters compare control and salicylic acid treatment within each water level (Tukey’s test; *p* ≤ 0.05). (**a**) chlorophyll *a*; (**b**) chlorophyll *b*; (**c**) chlorophyll *a/b*; (**d**) total carotenoids.

Drought stress increased the concentration of chlorophyll *a*, chlorophyll *b*, and carotenoids compared to the well-watered condition; however, SA application reversed this effect, reducing the concentration of pigments compared to the control (Figure 3a,b,d). By contrast, SA increased the concentration of chlorophyll *a*, chlorophyll *b*, and carotenoids compared to the control in re-watered plants (Figure 3a,b,d). Moreover, within SA-treated plants, re-watering resulted in the highest concentration of pigments compared to well-watered and drought conditions. Re-watering reduced the chlorophyll *a*/*b* ratio in control plants compared to well-watered plants (Figure 3c). In turn, SA reduced the chlorophyll *a*/*b* ratio in well-watered plants but increased that of re-watered plants, compared to the control (Figure 3c).

**Figure 4:**
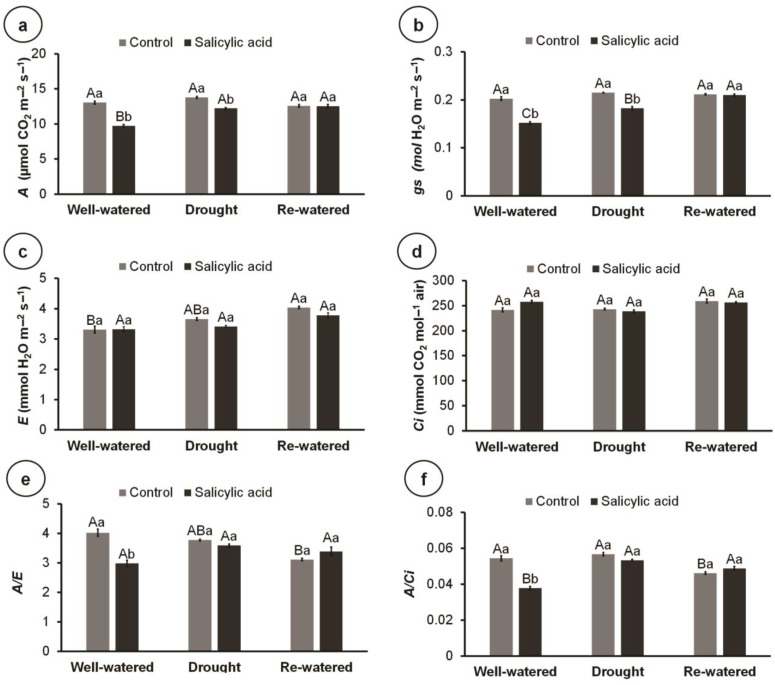
Gas exchange parameters of 100-day-old culantro plants treated with water (control) or salicylic acid and grown under different water levels (80% BC—well-watered; 40% BC—drought; and re-watered after 12 days of water restriction). Values represent means ± standard error (*n* = 6). Capital letters compare water levels within each salicylic acid treatment, and lowercase letters compare control and salicylic acid treatment within each water level (Tukey’s test; *p* ≤ 0.05). (**a**) Net carbon assimilation rate (*A*); (**b**) stomatal conductance (*g_S_*); (**c**) leaf transpiration rate (*E*); (**d**) internal CO_2_ concentration (*Ci*); (**e**) water use efficiency (*A*/*E*); (**f**) instantaneous carboxylation efficiency (*A*/*Ci*).

Within control plants, *A*, *g_S_*, and *Ci* were not affected by water conditions; however, re-watered plants had higher *E*, and lower *A*/*E* and *A*/*Ci* than well-watered ones (Figure 4a–f). On the other hand, SA application reduced A and *g_S_* compared to control plants under well-watered and drought conditions (Figure 4a,b). Similarly, SA reduced *A*/*E* and *A*/*Ci* under well-watered conditions (Figure 4e,f). Interestingly, *E* was not affected by SA treatment, while *Ci* was not affected by either the water condition or SA treatment (Figure 4c,d).

**Figure 5:**
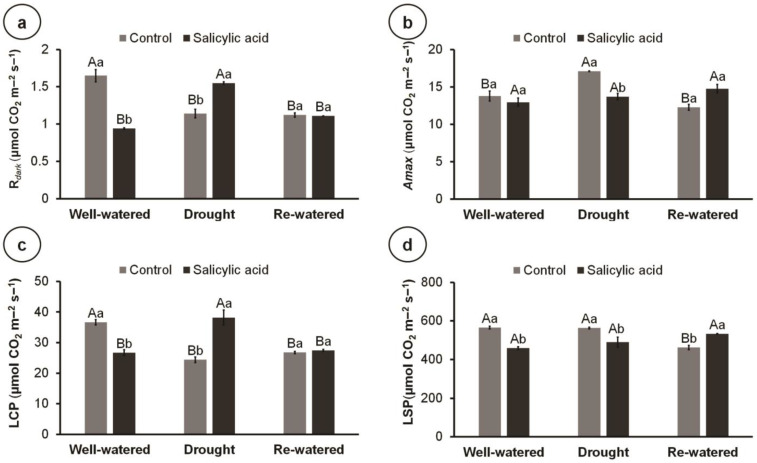
Light-curve parameters of 100-day-old culantro plants treated with water (control) or salicylic acid and grown under different water levels (80% BC—well-watered; 40% BC—drought; and re-watered after 12 days of water restriction). Values represent means ± standard error (n = 6). Capital letters compare water levels within each salicylic acid treatment, and lowercase letters compare control and salicylic acid treatment within each water level (Tukey’s test; *p* ≤ 0.05). (**a**) Dark respiration (R_dark_); (**b**) maximum gross assimilation rate (A_max_); (**c**) light compensation point (LCP); (**d**) light saturation point (LSP).

There was a reduction in the R_dark_ and LCP of control plants under drought stress; however, SA reversed this effect, increasing R_dark_ and LCP under drought (Figure 5a,c). Similarly, re-watering reduced the R_dark_ and LCP of control plants compared to the well-watered condition; however, SA did not affect these variables under the re-watered condition (Figure 5a,c). On the other hand, drought increased the A_max_ of control plants compared to well-watered and re-watered conditions (Figure 5b). Moreover, A_max_ was reduced by SA under drought compared to control plants. Within control plants, re-watering resulted in the lowest LSP, while no differences between water conditions were found within SA-treated plants (Figure 5d). Furthermore, SA reduced LSP under well-watered and drought conditions compared to control plants, while under the re-watered condition, SA increased LSP (Figure 5d).

**Figure 6:**
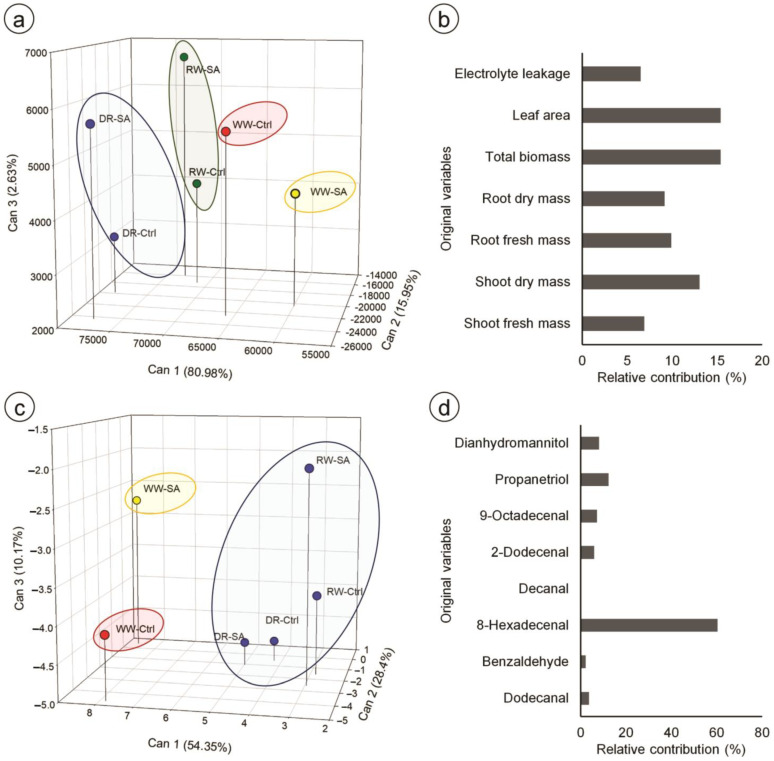
Canonical variables from original parameters in culantro plants treated with water (control) or salicylic acid and grown under different water levels (80% BC—well-watered; 40% BC—drought; and re-watered after 12 days of water restriction). (**a**,**c**) 3D scatter plots of the first three canonical components obtained from the morphophysiological variables and essential oil profile, respectively; (**b**,**d**) relative contribution of original variables to the canonical variables, calculated using the Singh method. The percentage of total variance explained by each canonical component is indicated in parentheses; treatments into the same ellipses were grouped by the Tocher optimization method and the generalized distance of Mahalanobis.

The first three canonical variables explained 99.56% of the variability among the treatments for morphophysiological parameters, allowing for a three-dimensional scatter plot representation (Figure 6a). The treatments were separated into four groups: group 1 (blue circle), comprising control and SA-treated plants under drought (DR-Ctrl and DR-SA); group 2 (green circle), comprising control and SA-treated plants under re-watered condition (RW-Ctrl and RW-SA); group 3 (red circle), comprising well-watered control plants (WW-Ctrl); and group 4 (yellow circle), comprising well-watered plants treated with SA (WW-SA). The relative contributions of the original variables showed that leaf area (15.4%), total biomass (15.4%), and shoot dry mass (13%) were the variables that most contributed to the total variance (Figure 6b).

Regarding the essential oil profile, the first three canonical variables explained 92.92% of the variability among treatments (Figure 6c). Treatments were separated into three groups: group 1 (red circle), corresponding to well-watered control plants (WW-Ctrl); group 2 (yellow circle), corresponding to well-watered plants treated with SA; and group 3 (blue circle), comprising control and SA-treated plants under drought stress and re-watered conditions (DR-Ctrl, DR-SA, RW-Ctrl, and RW-SA). The relative contributions of the original variables showed that 8-hexadecenal was the compound that most contributed to the total variance (60.3%) (Figure 6d).

## 4. Discussion

Water is essential for plant growth, and drought stress represents a major challenge for agriculture. Drought stress impacts crop production by affecting plant metabolism, physiology, and biochemistry [19,38]. Our results showed that drought stress reduced the leaf area of culantro plants but did not decrease biomass production and the number of leaves. Drought is known to reduce cell turgor pressure, inhibiting cell expansion and, consequently, plant growth [39,40]. This may explain the reduction in the leaf area of culantro under drought stress; however, no reduction in biomass production was found under drought, suggesting that culantro might be tolerant to irrigation with 40% BC. Interestingly, re-watering plants after 12 of water restriction was the condition that resulted in the highest leaf and root biomass, number of leaves, and leaf area. These results suggest that water restriction might have primed culantro plants, inducing defense responses that resulted in better performance after re-watering. In fact, studies have shown that drought stress priming may improve drought tolerance in plants due to “stress memory” mechanisms, such as the maintenance of water status, osmotic adjustment, and the expression of stress-related genes [41,42].

Under drought stress, plants generally direct more biomass to the roots, increasing their ability to absorb water and nutrients [43,44]. Here, water conditions did not alter the biomass partitioning of control plants, but SA treatment increased biomass allocation to shoots under the well-watered condition, as shown by the higher shoot/root ratio. Moreover, SA treatment strongly increased leaf and root biomass under the well-watered condition, indicating the role of this plant hormone in plant growth regulation. Indeed, SA has been related not only to stress responses but also to growth regulation [45,46]. Considering that the organ of commercial interest for culantro is the leaf [26], our results indicate that SA treatment has the potential to increase the commercial production of culantro under well-watered conditions. In the same way, re-watering increased overall culantro growth independently of SA treatment, suggesting that drought priming is a promising technique to increase biomass production in culantro. 

The specific leaf area is an estimative of leaf thickness that plays an important role in the ecological characteristics of plants, explaining variations in photosynthetic and respiratory rates per unit of leaf dry mass and in light interception [47,48,49]. Here, water conditions did not affect specific leaf area in control plants; however, in SA-treated plants, re-watering resulted in the highest specific leaf area, indicating that this condition resulted in thinner leaves. As increased specific leaf area upon SA treatment has been related to the role of this hormone in nutrient uptake and to the crosstalk with ethylene [50,51], this could help to explain the higher specific leaf area under this condition. 

Electrolyte leakage is an indicator of cell membrane integrity, with increases in electrolyte leakage values indicating higher leakage of ions due to a loss of membrane integrity [52]. In plant cells, electrolyte leakage can be detected almost instantly after the exposition to a stress factor due to K^+^ efflux and the production of ROS, which can cause oxidative damage to cell membranes and impair plant development [53,54]. SA application did not reduce but even increased electrolyte leakage in drought-stressed culantro plants, indicating reduced membrane stability. Considering that the effectiveness of SA in stress mitigation is highly dependent on its concentration and that high levels of SA can induce oxidative damage [22,55], our results suggest that SA treatment, combined with drought, may have induced oxidative damage in culantro. By contrast, re-watering resulted in the lowest electrolyte leakage independently of SA addition, indicating higher membrane integrity. Considering that plants induce antioxidant defenses in response to drought, reducing oxidative damage and increasing membrane stability [56], our results suggest that drought priming may have induced such responses in culantro plants, explaining the reduction in electrolyte leakage upon re-watering observed here. Recovery after a period of stress is a key mechanism in drought tolerance and plant survival, especially in regions where plants are exposed to repeated cycles of drought and irrigation [57]. Thus, our results further confirm the beneficial effect of drought priming in culantro plants. 

SA has been related to increases in the content of chlorophylls and carotenoids and reductions in chlorophyll catabolism [58,59]. Here, SA increased the content of chlorophyll *a*, chlorophyll *b*, and carotenoids in re-watered plants and reduced them in drought-stressed plants. These results, together with electrolyte leakage results, suggest that the combination of drought stress and SA treatment may have triggered oxidative damage, degrading photosynthetic membranes and pigments. By contrast, in re-watered plants, drought priming may have induced antioxidant defenses, which in combination with SA, even increased pigment contents and chloroplast membrane stability. In fact, SA has been related to changes in the monogalactosyldiacylglycerol/digalactosyldiacylglycerol ratio in chloroplast membranes, increasing the stability of photosystem complexes and chloroplasts [60,61]. Accordingly, SA treatment has also been shown to reduce electrolyte leakage and increase chlorophyll content in other species, such as *Linum usitatissimum*, under abiotic stresses [62].

Exogenous SA has been shown to affect the photosynthetic capacity of plants, depending on factors such as plant species, duration of treatment, form of application, and environmental conditions, with high SA concentrations negatively affecting photosynthesis [17]. This may explain the reduction in the photosynthetic capacity of plants treated with SA under well-watered and drought conditions. Furthermore, SA treatment decreased the *g_S_* of well-watered and drought-stressed plants, suggesting that here there was no antagonism between SA and abscisic acid; however, *Ci* remained high under these conditions, indicating that the lower *A* was not related to the CO_2_ availability [63]. In turn, the lower carboxylation efficiency (*A*/*Ci*) of well-watered plants treated with SA suggests that this treatment may have affected biochemical reactions. In fact, SA has been related to changes in biochemical reactions and the composition and volume of thylakoid membranes, decreasing photochemical efficiency and photosynthetic rates [63,64].

Dark respiration is a redox process, where the amount of CO_2_ released by respiration is greater than the CO_2_ fixed in photosynthesis [65]. Here, drought stress and re-watering reduced R_dark_ and LCP in control plants, and SA reversed this effect under drought, increasing R_dark_ and LCP. These results indicate that SA-treated plants had higher energy consumption and, thus, required higher light quantities to compensate for the levels of CO_2_ uptake released by plants through respiration and photosynthetic processes. Similar results were observed in *Arabidopsis* mutants with high SA content, which also exhibited increased dark respiration [66]. SA also reversed the increased A_max_ in drought plants and reduced LSP in drought and well-watered plants, suggesting that SA may be inducing photoinhibition under these conditions. Considering that photoinhibition is associated with oxidative damage in chloroplast membranes [67], these results further confirm the hypothesis that SA treatment may have induced oxidative stress, dismantled membranes, and increased electrolyte leakage. 

Canonical analysis showed that SA altered the morphophysiology of well-watered plants; however, under drought and re-watering conditions, the morphophysiological responses of the control were grouped with those of SA-treated plants. This indicates that the water condition was the main factor involved in the range of morphophysiological responses. Regarding the essential oil profile of culantro, our results indicated that water stress and SA application affected the essential oil profile. Due to its action in plant defense responses, SA is largely known as an elicitor of secondary metabolite production in plants [68,69]. Accordingly, our results showed that SA treatment altered the essential oil profile of well-watered plants; however, drought and re-watering also altered the essential oil profile of culantro independently of SA. These results indicate that the secondary metabolites produced in response to water stress are different from those produced under well-watered or SA treatments. In fact, it is known that the synthesis of secondary metabolites is highly affected by environmental factors, especially water stress, altering metabolite composition and yield [70]. Among the compounds found here, 8-hexadecenal, also known as trogodermal, was the compound that contributed most to the total variance. This compound was also identified as one the main constituents of essential oils in *Foeniculum vulgare* [71] and is characterized as a highly active sexual pheromone of Trogoderma, leading them to a mating behavior [72,73,74]. These findings contribute to future studies aimed at increasing the production of compounds of interest.

## 5. Conclusions

Drought reduced the leaf area, which was recovered upon rehydration. Moreover, re-watering caused a drought-priming effect that resulted in increased growth and membrane stability. The exogenous application of salicylic acid enhances growth and modulates the essential oil profile in well-watered culantro. The composition of essential oils was also modulated by water stress, whether followed by rehydration or not. Understanding the regulation of the profile of essential oils in culantro may enable the production of compounds of economic and industrial interest. Thus, our results generate new perspectives to explore the production of compounds of interest in culantro essential oils.

## Data Availability

The raw data supporting the conclusions of this article will be made available by the corresponding author upon reasonable request.
