# Correction: Santos et al. Salicylic Acid and Water Stress: Effects on Morphophysiology and Essential Oil Profile of *Eryngium foetidum*. *Metabolites* 2024, *14*, 241

**DOI:** 10.3390/metabo16080520

**Published:** 2026-07-24

**Authors:** Sabrina Kelly dos Santos, Daniel da Silva Gomes, Vanessa de Azevedo Soares, Estephanni Fernanda Oliveira Dantas, Ana Flávia Pellegrini de Oliveira, Moises Henrique Almeida Gusmão, Elyabe Monteiro de Matos, Tancredo Souza, Lyderson Facio Viccini, Richard Michael Grazul, Juliane Maciel Henschel, Diego Silva Batista

**Affiliations:** 1 Postgraduate Program in Agronomy, Federal University of Paraiba, Areia 58397-000, Paraíba, Brazil; sabrinasks11@gmail.com (S.K.d.S.);; 2 Department of Agriculture, Federal University of Paraiba, Bananeiras 58220-000, Paraíba, Brazil; 3 Department of Chemistry, Federal University of Juiz de Fora, Juiz de Fora 36036-900, Minas Gerais, Brazil; 4 Department of Biology, Federal University of Juiz de Fora, Juiz de Fora 36036-900, Minas Gerais, Brazil; 5 Postgraduate Program in Agroecology, Federal University of Paraiba, Bananeiras 58220-000, Paraíba, Brazil

The authors would like to make the following correction to their published paper [1]. The change is as follows:

Reference [54] in the original publication was retracted after our article had already been published. As the retraction occurred after publication, the reference was considered valid at the time the manuscript was prepared and published. Upon becoming aware of its retracted status, we decided to replace it with the following reference, which provides appropriate support for the statement in question:

54. Dhiman, S.; Sharma, P.; Bhardwaj, T.; Devi, K.; Khanna, K.; Kapoor, N.; Kaur, R.; Sharma, A.; Kaur, R.; Bhardwaj, R. Role of Potassium in Heavy Metal Stress. In *Role of Potassium in Abiotic Stress*; Iqbal, N., Umar, S., Eds.; Springer: Singapore, 2022. https://doi.org/10.1007/978-981-16-4461-0_8.

The authors state that the scientific conclusions are unaffected. This correction was approved by the Academic Editor. The original publication has also been updated.
